# Perceived effectiveness and preferences of medical students toward blended learning in anatomy: a multi-institutional cross-sectional study

**DOI:** 10.1186/s12909-025-08336-8

**Published:** 2025-12-06

**Authors:** Ehab Mostafa Elzawawy, Hassan Reda Hassan Elsayed, Marwa Mahmoud Mady, Mennatallah H. Rizk, Saleh Riyadh Almuktar, Abdullah Kamal Al-hajjar, Karim Ahmed Alsherbini, Melad N. Kelada

**Affiliations:** 1 Department of Human Anatomy and Embryology, Faculty of medicine, Alexandria University, Alexandria, Egypt; 2 Department of Human Anatomy and Embryology, Faculty of medicine, Mansoura University, Mansoura, Egypt; 3 Biomedical Sciences Department, College of Medicine, Gulf Medical University (GMU), Ajman, UAE; 4 Medical education department, Faculty of Medicine, Alexandria University, Alexandria, Egypt; 5 Department of Anatomy and Neurobiology, College of medicine and health sciences, National University, Sohar, Oman

**Keywords:** Blended learning, Anatomy education, Self-regulated learning, Small groups activities, Moodle, Face to face learning, Online learning

## Abstract

**Background:**

The rapid integration of blended learning (BL) into anatomy education has transformed traditional teaching. the preferences and perceptions of medical students toward BL, and its impact on anatomy learning, remain underexplored.

**Objective:**

This multi-institutional study aimed to assess medical students’ preferences and perceptions regarding BL in anatomy education, and to identify factors influencing their anatomy learning across three universities in Egypt and Oman.

**Methods:**

A comparative cross-sectional survey was conducted among 615 medical students from Alexandria University (Egypt), Mansoura University (Egypt), and National University (Oman). The validated Blended Learning Questionnaire (BLQ), adapted from Western Sydney University, was administered online. The BLQ evaluated preferences for learning modalities, satisfaction with BL, the role of self-regulated learning (SRL), small group activities. Data were analysed using descriptive statistics, chi-square tests, t-tests, and ANOVA, with significance set at *p* < 0.05.

**Results:**

students expressed a preference for BL and online modalities over traditional face-to-face lectures, with the highest preference for BL observed in National University. Female students favoured small group learning, while SRL was most valued by National students. The use of audio-visual resources and flexibility in accessing online materials were highly rated. Institutional differences were noted in preferred online tools and the value of small group activities.

**Conclusion:**

Medical students across diverse settings prefer blended and online learning approaches for anatomy, highlighting the need for flexible, resource-rich, and student-cantered curricula. These highlight the importance of supporting SRL and using technology to optimize anatomy education, with implications for curriculum design and faculty development.

## Introduction

The introduction of blended learning (BL) into medical education has gone through many levels in the past two decades. The combination of face-to-face instructions and online learning is referred to as BL [1, 2]. It has substituted the traditional face-to-face lectures in many medical schools [3]. The integration of variable learning methods to help students achieve their learning outcomes has seen many pedagogical adaptations [4]. This approach enhances the development of a more student-centered learning which helps them to mature into lifelong learners. Nevertheless, the impact of these curricular changes on student learning behavior and achievement has not been well investigated [5].

In BL, the learning process is dynamic and not static as in traditional learning. This dynamic learning process is initiated by the learner. This dynamic student participation ensures that they are continuously evaluating their success level and can change their strategies accordingly. This dynamic BL will evolve eventually into Self-directed learning (SDL) and Self-regulated learning (SRL) [6].

SDL and SRL are often used interchangeably in literature. The most substantial difference is that in SDL the student should have a control over the external learning environment, while the main focus of SRL is internal, where the students set up their goals, plan, use and adjust strategies to accomplish these goals; manage available resources, control their emotions, cognition and behaviour to attain these goals and self-evaluate whether they have achieved their goals or not and modify or adapt new strategies for future learning thus reflecting on their progression [7, 8]. Medical practitioners need to keep up with the latest updates, recommendations and guidelines and thus SRL becomes essential for successful professional development [9].

This process can be challenging and depend largely on the available social support and the surrounding learning environment. These may, in turn, affect the learners’ level of success. Therefore, medical schools must take the initiative to provide online learning resources [10].

During the COVID pandemic, the role of online learning became clear and ever since, BL was used more broadly in medical education [11]. Many educational theories emphasize the importance of BL and SRL on student learning behavior and help the educators improve the delivery of the course material [12].

The Motivated Strategies for Learning Questionnaire (MSLQ) has been used to evaluate SRL since 1991 [13]. It can be used to assess the relationship between the learner and the educator and evaluate learning techniques and strategies in SRL [14]. Medical education unit in west Sydney Australia developed a validated Questionnaire to assess the effect of medical students’ SRL in the BL environment. They called it blended learning questionnaire (BLQ). Valuable data can be obtained from this BLQ including students’ learning behaviors, preferred method of learning and this can help educators to assess curricula, modify content delivery and adjust the learners’ environment [15].

Despite the increasing adoption of blended learning (BL) in medical education and the recognized importance of self-regulated learning (SRL), small group learning, and external resources, there remains a lack of comprehensive understanding of how these factors interact and vary across diverse institutional and cultural contexts. Existing studies have primarily focused on single institutions or specific components of learning preferences without systematically comparing multi-institutional cohorts across different countries.

Furthermore, validated instruments to explore medical students’ learning behaviors in blended environments are limited, and the influence of demographic factors such as gender and university culture on learning preferences is underexplored. This gap hinders the development of tailored, evidence-based educational strategies that optimize student engagement and academic outcomes in varied settings.

The main aim of this study is to comprehensively explore and compare medical students’ learning preferences, behaviours, and motivational factors within blended learning environments across three universities in Egypt and Oman. Specifically, the study seeks to assess students’ preferences for online, face-to-face, and blended learning modalities; evaluate the perceived importance of self-regulated learning, small group learning, and external resources; and investigate the influence of gender and institutional context on these preferences.

To achieve this, the research objectives include: (1) identifying predominant learning modality preferences among students at Alexandria, Mansoura, and National universities; (2) examining students’ engagement with self-regulated learning and goal-setting strategies; (3) analysing gender differences in learning preferences and behaviours; and (4) understanding the role of external and institutional resources in supporting anatomy education.

The study is guided by key research questions: What are the variations in learning modality preferences across the three universities? How do students perceive the impact of motivation, goal setting, and self-regulation on their learning? Are there significant gender-based differences in learning preferences? And how do institutional factors shape these educational experiences? Addressing these questions fills a critical gap in medical education research by providing multi-institutional, cross-cultural insights that can inform tailored curricular innovations and enhance student-cantered learning strategies in diverse educational settings.

## Materials and methods

### Study design and setting

This study utilized a descriptive, comparative, cross-sectional survey design to assess undergraduate medical students’ experiences across multiple institutions in the period between January and May 2024. A survey methodology was selected to efficiently gather standardized data from a large, geographically dispersed student population, enabling comparison across different universities. Data was collected by visiting the colleges and through *Google forms* (Google LLC, California, USA) from students exposed to blended learning.

### Study participant and sampling

The target population included undergraduate medical students enrolled from Year 1 to Year 5 at Alexandria University (Egypt), Mansoura University (Egypt), and National University (Oman). Convenience sampling was utilized to recruit participants accessible during the study period. Only medical students, affiliated with the institutions mentioned, have been exposed to both learning modalities; face to face and online, with sufficient language proficiency and voluntarily consent to share in the study. No formal power analysis was conducted; however, the sample size was considered sufficient for descriptive and comparative analyses.

### Data collection tool and technique

We used an adopted blended learning questionnaire (BLQ) to explore the preferred learning method for studying Anatomy from the perspective of the students and how much they are satisfied with different learning modalities. This BLQ was adopted from the medical education unit in west Sydney University (WSU) Australia [14].

### BLQ validation

The Blended Learning Questionnaire (BLQ) was created and validated by the Medical Education Unit at Western Sydney University (WSU). Minor modifications were made to align it with the educational context of the participating universities and anatomy courses. The validation process was carried out in two consecutive stages, and the details of the modified items are presented in Table 1.

**Table 1.** Comparison of survey items – adopted BLQ used in current study and original BLQ

Item No.	Adapted BLQ used in current study	Original BLQ [12]	Dissimilarities/Notes
1	I actively seek online resources to prepare my learning materials before a learning activity…	Identical	No difference
2	I find small group work enhances my understanding about a particular concept	Identical	No difference
3	I am able to strengthen my learning following a small group activity	I am able to consolidate my learning following a small group activity	“strengthen” vs. “consolidate” slight wording change, but meaning is similar
4	I find external audio-visual online resources important to my learning	I find external audio-visual online resources very important to my learning	Added emphasis: “very important” in original BLQ.
5	I find the audio-visual online resources provided by the School of Medicine crucial for my learning	Identical	No difference
6	Flexibility to use a variety of online material motivates my independent learning.	Identical	No difference
7	The way I utilize study materials changes as exams approach	My use of study resources differs leading up to exams.	Different phrasing, but similar meaning
8	My motivation to study increases leading up to exams.	Identical	No difference
9	My study is stimulated by group discussions	Identical	No difference
10	My study habits are influenced by my peers/social interactions.	My study habits are influenced by my peers/social interaction.	Minor difference: “interactions” (plural) vs. “interaction” (singular)
11	I set up study goals that organize/structure my learning	I set up study goals that organize/structure my learning	Minor spelling difference: “organize” (US) vs. “organize” (UK)
12	My study is influenced by the fact that I need to maintain my image (among peers/supervisors)	Identical	No difference
13	Accessibility to School of Medicine lectures online enhances my independent learning.	Identical	No difference
14	I learn more efficiently when I’m able to access online resources using different devices	Identical	No difference
15	Access to online material off-campus enables me to structure my independent learning	Identical	No difference
16	I use School of Medicine lecture material as a guide for what to learn	Identical	No difference
17	Some online resources are efficient because they are well summarized	Some online resources are efficient because they are well summarized	Minor spelling difference: “summarized” (US) vs. “summarized” (UK)
18	Specific external online resources are vital to my independent learning	Identical	No difference
19	I often integrate a variety of medical school and external online resources to support my learning	Identical	No difference

#### First stage – Student validation (face and contextual validity)

Sixty medical students in the preclinical phase (twenty from each university) were involved. They represented different ages, genders, and ethnicities to ensure demographic diversity. Focus group discussions (FGDs) were conducted voluntarily and anonymously. Students reviewed each question for clarity and contextual relevance, discussed emerging themes, and assisted in refining the language of the items. They then participated in pilot testing to ensure comprehension and usability of the adapted version. Feedback from this stage was incorporated to generate the final questionnaire version summarized in Table 1.

#### Second stage – Expert validation (content validity)

Content and face validity were confirmed by a panel of 12 experts in medical education and allied health from Alexandria, Mansoura, and National Universities. The experts assessed the relevance and clarity of each item using a 3-point scale (essential = 3, useful but not essential = 2, not necessary = 1). The Content Validity Ratio (CVR) for each item was calculated using the formula:

### CVR = (Nₑ - N/2)/(N/2)

where Nₑ is the number of experts rating the item as essential and N is the total number of experts. Items with a CVR value below 0.59 were excluded (none were removed). The overall Content Validity Index (CVI) for the questionnaire was 0.856, indicating excellent content validity.

### Reliability testing

Pilot testing confirmed that all items were clear and consistent. The adopted BLQ contained 19 items, each rated on a 4-point Likert scale (1 = not at all true of me to 4 = very true of me), simplified from the original 7-point scale to enhance feasibility and response clarity. The instrument demonstrated satisfactory internal consistency, with a Cronbach’s alpha of 0.75, confirming reliable measurement across scales.

The BLQ was transformed into an online form through Google Forms and shared with the students during lectures. The purpose of the study was explained to students and confidentiality was assured, following which they were asked for their voluntary participation.

The link was disabled after 10 days of circulating the Google form. Data were collected regarding demographic variables, acceptance, preferences, perception, pros and cons, limitations and suggestions for online teaching.

The survey items were systematically categorized into two complementary frameworks to gain deeper insights into students’ learning preferences and behaviours. First, the items were classified according to their alignment with different learning modalities: those favouring face-to-face learning (items 2, 3, 9, 10, 12), those favouring online learning (items 4, 5, 6, 13, 14, 15, 18), and those favouring blended learning (items 1, 7, 16, 17, 19). This classification underscores the diversity of students’ preferences across instructional formats [Table 2].

**Table 2.** Distribution of adopted BLQ survey items into two categories

Category	Question Numbers
Learning modalities	
Favoring Face-to-Face Learning	2, 3, 9, 10, 12
Favoring Online Learning	4, 5, 6, 13, 14, 15, 18
Favoring Blended Learning (BL)	1, 7, 16, 17, 19
Factors influencing student learning	
Small Group Learning	2, 3, 9, 10
Impact of Goal Setting on Learning	8, 11, 12
Importance of SRL	1, 5, 6, 7,13, 14, 15
Influence of External Resources	4, 16, 17, 18, 19

Second, the items were grouped based on key learning factors influencing student engagement: small group learning (items 2, 3, 9, 10), the impact of goal setting on learning (items 8, 11, 12), the importance of self-regulated learning (SRL) (items 1, 5, 6, 7, 13, 14, 15), and the influence of external resources on learning (items 4, 16, 17, 18, 19). This dual categorization framework provides a nuanced understanding of how specific learning strategies and environmental factors contribute to students’ academic success across various learning environments [Table 2].

### Ethical considerations

This research was approved by the ethics and biosafety committee at the college of Medicine and Health sciences, National University for science and technology, Sohar, Oman. (registration number: NU/COMHS/EBC0018/2022). The research was approved by ethics committee of Faculty of Medicine, Alexandria University, Alexandria, Egypt. [IRB No: 00012098 - FWA No: 00018699] (serial number:0306374).

Informed consent was obtained from all participants, emphasizing voluntary participation, confidentiality, and the right to withdraw at any time. Data privacy was maintained by anonymizing responses and securely storing data in password-protected systems, adhering to institutional and international ethical standards. 

### Statistical analysis

Data were fed to the computer using IBM SPSS software package version 24.0. Qualitative data were described using number, frequency and percentage. Comparison between different groups regarding categorical variables was tested using Chi-square test. Quantitative data were described using mean and standard deviation for normally distributed data. For normally distributed data, comparison between two independent populations was done using independent t-test, while comparing between more than two groups ANOVA test was used. The significance of the results obtained was at the 5% level. All the statistics were significant when *p* < 0.05. Data was presented in the form of tables.

## Results

This study conducted a multi-institutional assessment of blended learning (BL) preferences among medical students from three universities: Alexandria and Mansoura in Egypt, and National University in Oman. A total of 615 medical students participated, including 279 (75% of all enlisted students in anatomy courses) from Alexandria University, 101 (72% of all enlisted students in anatomy courses) from Mansoura University, and 235 (80% all enlisted students in anatomy courses) from National University. The participants’ ages ranged from 17 to 27 years. At Alexandria University, the majority of students were aged 17–20 years, whereas Mansoura University students were predominantly in the 20–25 age group. At National University, the student population was almost evenly split between the 17–20 and 20–25 age groups. Regarding gender distribution, Alexandria had a slightly higher proportion of females, with a male-to-female ratio of 1:1.14. Mansoura University had more males, with a ratio of 1.4:1, while at National University, females constituted the majority, accounting for 78.3% of the participants, as detailed in Table 3.

**Table 3.** Distribution of the studied group regarding their demographic data

	Alexandria University “*n* = 279”		Mansoura University “*n* = 101”		National University “*n* = 235”		Total
Age	No.	%	No.	%	No.	%	
17-<20	186	66.7	12	11.9	121	51.5	319
20-<25	88	31.5	70	69.31	113	48.1	271
25–27	5	1.8	19	18.81	1	0.4	25
Range	18.0–25		17.0–27.0		18.0–26.0		
Mean ± S.D.	19.5 ± 1.91		23.6 ± 2.5		20.8 ± 2.1		
Gender	No.	%	No.	%	No.	%	Total
Male	130	46.6	59	58.4	51	21.7	240
Female	149	53.4	42	41.6	184	78.3	375

A comparative analysis was conducted to examine the learning modality preferences—online, face-to-face, and blended learning—among medical students from Alexandria, Mansoura, and National Universities, revealing notable differences across the three institutions as presented in Table 4. The chi-square tests reveal statistically significant differences in learning modality preferences both overall and between universities. Specifically, a high proportion of students from National University (92.3%) preferred online learning compared to Alexandria (80.6%) and Mansoura (76.2%) (χ²=19.342, *p* = 0.0012). Similarly, face-to-face learning was favoured more by National University students (82.1%) than those from Alexandria (55.2%) and Mansoura (63.3%) (χ²=18.692, *p* = 0.0016). Blended learning showed a similar pattern, with National University students again reporting the highest preference (91.4%), followed by Alexandria (80%) and Mansoura (78.2%) (χ²=15.811, *p* = 0.0019) [Table 4].

**Table 4.** Comparison of learning modality preferences among students from Alexandria, Mansoura, and National Universities

Learning modalities	Alexandria University “*n* = 279”		Mansoura University “n101”		National University “235”		Total *N* = 615	X^2a^ *P*	P1 P2 P3
	No	%	No	%	No	%			
Online Learning	225	80.6	77	76.2	217	92.3	519	19.342 0.0012*	0.347 0.002* 0.0013*
Face to face	154	55.2	64	63.3	193	82.1	411	18.692 0.0016*	0.154 0.002* 0.004*
Blended learning	223	80	79	78.2	215	91.4	517	15.811 0.0019*	0.715 0.002* 0.001*
X^2b^	58.02		6.604		43.16				
P	0.001*		0.036*		0.001*				
P1	0.001*		0.046*		0.001*				
P2	0.831		0.737		0.735				
P3	0.001*		0.020*		0.001*				

a=comparison between the three universities regarding the type of learning

b=comparison between the types of learning in the same university

Pairwise comparisons (P1, P2, P3) indicate significant differences between specific university pairs in most cases, particularly involving National University, which consistently shows higher preference percentages. The overall chi-square values for each learning modality (X2b) further support significant associations between university affiliation and learning preferences (all *p* < 0.05). These results suggest that students’ preferences for instructional formats vary significantly by university, with National University students demonstrating stronger preferences across all three modalities. This may reflect differences in institutional resources, teaching approaches, or student demographics. The significant chi-square values and low p-values confirm that these observed differences are unlikely due to chance, indicating a meaningful association between university and learning modality preference [Table 4].

Mean scores and standard deviations (SD) illustrating students’ preferences for three learning modalities—online, face-to-face, and blended learning—were analysed across three universities. Analysis of variance (ANOVA) tests were performed to assess differences in mean preference scores among the institutions as presented in Table 5.

For online learning, National University students reported the highest mean preference score (M = 3.14, SD = 0.49), followed by Alexandria (M = 2.92, SD = 0.62) and Mansoura (M = 2.82, SD = 0.50). The overall ANOVA indicated a statistically significant difference among the universities (F = 15.1, *p* = 0.001). Pairwise comparisons revealed significant differences particularly between National and Mansoura (*p* = 0.002) and National and Alexandria (*p* = 0.01), suggesting National University students have a stronger preference for online learning [Table 5].

Regarding face-to-face learning, National University again showed the highest mean preference (M = 2.81, SD = 0.60), with Mansoura (M = 2.71, SD = 0.48) and Alexandria (M = 2.54, SD = 0.61) following. The ANOVA test confirmed significant differences across universities (F = 13.75, *p* = 0.001). Pairwise tests indicated significant differences between Alexandria and Mansoura (*p* = 0.015) and between Alexandria and National University (*p* = 0.019), while the difference between Mansoura and National University was not statistically significant (*p* = 0.135) [Table 5].

**Table 5.** Comparison of student preferences for different learning modalities across three Universities based on mean scores

Learning modality	Alexandria University “*n* = 279”		Mansoura University “n101”		National University “235”		Total *N* = 615	ANOVA X^2a^ *P*	P1 P2 P3
	Mean	SD	Mean	SD	Mean	SD			
Online Learning	2.92	0.62	2.82	0.50	3.14	0.49	519	15.1 0.001*	0.11 0.01* 0.002*
Face to face	2.54	0.61	2.71	0.48	2.81	0.60	411	13.75 0.001*	0.015* 0.019* 0.135
Blended learning	2.84	0.59	2.82	0.44	3.09	0.47	517	16.150 0.001*	0.655 0.001* 0.001*
ANOVA X^2b^	16.52		12.25		11.9				
P	0.001*		0.005*		0.013*				
P1	0.002*		0.02*		0.036*				
P2	0.103		0.89		0.421				
P3	0.008*		0.021*		0.031*				

a=comparison between the three universities regarding the type of learning

b=comparison between the types of learning in the same university

For blended learning, National University students again reported the highest preference (M = 3.09, SD = 0.47), compared to Alexandria (M = 2.84, SD = 0.59) and Mansoura (M = 2.82, SD = 0.44). The ANOVA showed significant differences among the groups (F = 16.15, *p* = 0.001). Pairwise comparisons highlighted significant differences between National University and both Alexandria (*p* = 0.001) and Mansoura (*p* = 0.001), whereas Alexandria and Mansoura did not differ significantly (*p* = 0.655) [Table 5].

Overall, the ANOVA results (X2b) further confirmed significant differences in learning modality preferences among the three universities for online learning (*p* = 0.001), face-to-face learning (*p* = 0.005), and blended learning (*p* = 0.013). These findings suggest that students at National University consistently demonstrate stronger preferences for all three learning modalities compared to their counterparts at Alexandria and Mansoura Universities. The variation in preferences may reflect differences in institutional teaching methods, availability of resources, or cultural and educational contexts at each university [Table 5].

The distribution of participants preferred online tools for learning anatomy across three universities: Alexandria University (*n* = 279), Mansoura University (*n* = 101), and National University (*n* = 235), with a total of 615 respondents. The online tools considered include Telegram, WhatsApp, LMS Moodle, and a category labelled “Other,” which encompasses platforms such as YouTube, Google, online books, Instagram, and medical websites as presented in Table 6. The analysis reveals significant differences in tool preferences among the universities (χ² = 130.98, *p* = 0.001). The most preferred category overall was “Other,” selected by 61.6% of the total participants (379/615), with National University students showing the highest preference at 77.4%, compared to 53.8% at Alexandria and 46.5% at Mansoura. This suggests that students at National University rely more heavily on diverse online resources such as YouTube and medical websites for learning anatomy [Table 6].

**Table 6.** Distribution of the participants studied regarding the preferred online tools for learning anatomy across the three universities

Online tool	Alexandria University “*n* = 279”		Mansoura University “n101”		National University “235”		Total
	No	%	No	%	No	%	
Telegram	107	38.4	43	42.6	5	2.1	155
WhatsApp	22	7.8	0	0.0	17	7.2	22
LMS Moodle	0	0.0	11	10.9	31	13.3	59
Other^@^	150	53.8	47	46.5	182	77.4	379
X^2^	130.98						
P value	0.001*						
P1	0.073						
P2	0.002*						
P3	0.001*						

^@^Other:You tube, Google, online books, Instagram and medical websites

P1 comparison between Alexandria and Mansoura

P2 comparison between Alexandria and National

P3 comparison between Mansoura and National

Telegram was the second most popular tool, favored by 25.2% of all participants (155/615). Its use was notably high at Alexandria University (38.4%) and Mansoura University (42.6%), but very low at National University (2.1%). The pairwise comparisons indicate that the difference in Telegram usage between Alexandria and National University (*p* = 0.002) and between Mansoura and National University (*p* = 0.001) is statistically significant, while the difference between Alexandria and Mansoura is not (*p* = 0.073). This suggests that Telegram is a common communication and learning tool in the Egyptian universities but much less utilized by students at National University [Table 6].

WhatsApp was less commonly used overall, with only 6.2% of participants selecting it. Its use was concentrated in Alexandria (7.8%) and National University (7.2%) and completely absent in Mansoura (0%). The difference between Alexandria and National University in WhatsApp usage was not statistically significant, but the overall low usage suggests it is a less favored tool for anatomy learning among these groups [Table 6].

LMS Moodle showed moderate preference, particularly at National University (13.3%) and Mansoura University (10.9%), while Alexandria students did not report using it at all. The difference in Moodle usage between Alexandria and National University was statistically significant (*p* = 0.002), indicating that National University and Mansoura students are more engaged with formal learning management systems compared to Alexandria students [Table 6].

In summary, the findings highlight distinct preferences for online anatomy learning tools across the three universities. National University students predominantly use a broad range of external online resources (“Other”), while Alexandria and Mansoura students show higher reliance on messaging platforms like Telegram. The variation in tool preferences may reflect differences in institutional support, availability of platforms, or cultural and technological factors influencing students’ learning behaviors. These insights can inform targeted strategies to optimize online anatomy education tailored to each university’s context [Table 6].

The analysis of mean scores for student preferences regarding anatomy learning and teaching methods across Alexandria, Mansoura, and National universities reveals both similarities and notable differences in learning behaviors and resource utilization. Overall, students from all three universities reported moderate to high engagement with various learning strategies, with National University students consistently demonstrating slightly higher mean scores across most survey items, indicating stronger preferences or greater agreement Table 7. For instance, National University students showed the highest mean scores in actively seeking online resources before learning activities (M = 2.74), finding small group work beneficial (M = 2.85), and strengthening learning after group activities (M = 2.84), compared to Alexandria and Mansoura students, whose means ranged from 2.56 to 2.75. This suggests that National University students may be more proactive and collaborative in their learning approaches [Table 7].

**Table 7.** Mean scores of student preferences for anatomy learning and teaching methods at Alexandria, Mansoura, and National Universities (*N* = 615)

Survey items	Alexandria		Mansoura		National	
	Mean	SD	Mean	SD	Mean	SD
1. I actively seek online resources to prepare my learning materials before a learning activity (tutorial/lecture/ward presentation).	2.6	0.92	2.56	0.85	2.74	0.85
2. I find small group work enhances my understanding about a particular concept	2.6	0.86	2.65	0.87	2.85	0.78
3. I am able to strengthen my learning following a small group activity	2.6	0.86	2.75	0.70	2.84	0.67
4. I find external audio-visual online resources important to my learning	3.2	0.87	3.25	0.78	3.23	0.62
5. I find the audio-visual online resources provided by the School of Medicine crucial for my learning	2.6	0.97	2.39	0.84	3.06	0.68
6. Flexibility to use a variety of online material motivates my independent learning.	3.1	0.86	2.97	0.75	3.26	0.55
7. The way I utilize study materials changes as exams approach	2.9	0.92	2.96	0.79	3.07	0.66
8. My motivation to study increases leading up to exams.	2.9	0.99	3.09	0.85	3.01	0.74
9. My study is stimulated by group discussions	2.4	0.93	2.69	0.77	2.73	0.79
10. My study habits are influenced by my peers/social interactions.	2.4	0.94	2.65	0.81	2.74	0.81
11. I set up study goals that organize/structure my learning	2.9	0.87	2.88	0.77	3.03	0.68
12. My study is influenced by the fact that I need to maintain my image (among peers/supervisors)	2.7	0.91	2.79	0.71	2.70	0.79
13. Accessibility to School of Medicine lectures online enhances my independent learning.	2.6	0.96	2.38	0.88	3.05	0.69
14. I learn more efficiently when I’m able to access online resources using different devices	2.9	0.92	2.89	0.82	3.10	0.63
15. Access to online material off-campus enables me to structure my independent learning	2.9	0.90	2.79	0.74	3.12	0.60
16. I use School of Medicine lecture material as a guide for what to learn	2.7	0.96	2.37	0.88	3.27	0.62
17. Some online resources are efficient because they are well summarized	3.1	0.85	3.20	0.62	3.19	0.61
18. Specific external online resources are vital to my independent learning	3.1	0.90	3.12	0.77	3.08	0.62
19. I often integrate a variety of medical school and external online resources to support my learning	3.0	0.90	3.02	0.75	3.07	0.64

When considering the importance of audio-visual online resources, National University again scored highest in perceiving the School of Medicine’s resources as crucial (M = 3.06) and valuing flexibility in using various online materials (M = 3.26). Alexandria and Mansoura students showed moderate agreement but with lower means, particularly Mansoura students who reported the lowest mean (M = 2.39) regarding the importance of school-provided audio-visual materials. This pattern indicates that National University students may rely more heavily on institutional digital resources for their anatomy studies. Regarding study habits influenced by exams and motivation, all universities reported similar mean scores, with National University slightly ahead (means around 3.0), indicating a common trend of increased motivation and adaptive study strategies as exams approach. Group discussions and peer influence also showed National University and Mansoura students scoring higher (means ~ 2.7) than Alexandria students (~ 2.4), suggesting a stronger social component in their study habits [Table 7].

In terms of self-regulated learning factors, such as setting study goals and using online lectures to enhance independent learning, National University students again reported higher means (above 3.0), while Alexandria and Mansoura students showed moderate agreement (means between 2.37 and 2.9). Notably, National University students rated the use of lecture materials as a learning guide (M = 3.27) significantly higher than Mansoura (M = 2.37) and Alexandria (M = 2.7), highlighting a potentially more structured approach to learning [Table 7].

Across all universities, external online resources were consistently valued, with mean scores above 3.0, reflecting their vital role in supporting independent learning. Integration of various medical school and external resources also received positive ratings, with National University slightly leading. In summary, while students from Alexandria, Mansoura, and National University universities share common learning preferences and recognize the importance of online and group learning tools, National University students generally exhibit stronger engagement and preference for both institutional and external digital resources, as well as more proactive and structured learning behaviors. These differences may be influenced by variations in institutional support, technological infrastructure, or cultural factors affecting learning styles across the three universities [Table 7].

A comparative analysis of learning preferences between male and female medical students across four learning modalities: online learning, face-to-face learning, blended learning, and small group education presented in Table 8. Mean scores and standard deviations (SD) were calculated for each modality, and independent samples t-tests were performed to assess the statistical significance of gender differences.

**Table 8.** Comparative analysis of gender differences in learning preferences among medical students

Learning modality	Male	Female	T test *P* value
Online learning			
Mean	2.91	2.95	0.751
SD	0.60	0.522	0.387
Face to face learning			
Mean	2.67	2.7	0.298
SD	0.622	0.59	0.585
Blended learning			
Mean	2.93	3.03	1.36
SD	0.58	0.55	0.067
Small Group Education			
Mean	2.34	2.86	
SD	1.16	1.24	0.003*

*a statistically significant difference at the 0.005 level

For online learning, males and females showed very similar preferences, with mean scores of 2.91 (SD = 0.60) and 2.95 (SD = 0.52), respectively. The t-test result (t = 0.387, *p* = 0.751) indicates no significant difference between genders, suggesting that both male and female students equally value online learning.

Similarly, in face-to-face learning, males had a mean preference score of 2.67 (SD = 0.62) and females 2.70 (SD = 0.59), with a non-significant t-test value (t = 0.298, *p* = 0.585). This implies that gender does not significantly influence preferences for traditional face-to-face instructional methods [Table 8].

Regarding blended learning, females reported a slightly higher mean score (3.03, SD = 0.55) than males (2.93, SD = 0.58), but the difference was not statistically significant (t = 1.36, *p* = 0.067). Although females show a trend toward greater preference for blended learning, this difference does not reach the conventional threshold for significance [Table 8].

In contrast, small group education revealed a statistically significant gender difference. Female students reported a notably higher mean preference (2.86, SD = 1.24) compared to males (2.34, SD = 1.16), with the t-test indicating significance (t = 0.003, *p* < 0.005). This suggests that female students are more inclined to value small group learning environments, which may reflect differences in collaborative learning styles or social engagement preferences between genders [Table 8].

The distribution of medical students’ preferences regarding the importance of self-regulated learning (SRL) across three Universities presented in Table 9. Participants rated the extent to which SRL applies to them on a four-point scale ranging from “Not at all true of me” to “Very true of me.” The majority of students across all three universities acknowledged the importance of SRL, with 64.9% of Alexandria students, 70.3% of Mansoura students, and 74.0% of National University students responding, “True of me.” Additionally, the proportion of students who rated SRL as “Very true of me” was notably higher at National University (17.7%) compared to Alexandria (15.8%) and Mansoura (5.9%). Conversely, the percentages of students who disagreed with the importance of SRL (“Not at all true” and “Untrue of me”) were highest at Mansoura (23.8%) and Alexandria (19.4%), while National University reported the lowest disagreement (8.2%). The mean scores further illustrate these differences: National University students had the highest mean SRL preference (M = 3.1, SD = 0.4), followed by Alexandria (M = 2.9, SD = 0.60) and Mansoura (M = 2.82, SD = 0.49) [Table 9].

**Table 9.** Distribution and comparison of self-regulated learning (SRL) preferences among medical students at the three Universities

Importance of self-regulated learning (SRL)	Alexandria University “*n* = 279”		Mansoura University “n101”		National University “235”		Total
	No	%	No	%	No	%	
Not at all true of me	10	3.6	2	2.0	0	0.0	12
Untrue of me	44	15.8	22	21.8	19	8.2	85
True of me	181	64.9	71	70.3	171	74.0	423
Very true of me	44	15.8	6	5.9	45	17.7	95
Range	1–4		1.44–3.67		1.8–4		
Mean	2.9		2.82		3.1		
SD	0.60		0.49		0.4		
ANOVA test	12.134						
*P* value	0.001*						
P1	0.177 N.S.						
P2	0.001*						
P3	0.01*						

P1 comparison between Alexandria and Mansoura

P2 comparison between Alexandria and National

P3 comparison between Mansoura and National

The ANOVA test confirmed a statistically significant difference in SRL preference among the three universities (F = 12.134, *p* = 0.001). Pairwise comparisons revealed no significant difference between Alexandria and Mansoura (*p* = 0.177), but significant differences were found between Alexandria and National University (*p* = 0.001) and between Mansoura and National University (*p* = 0.01). These results indicate that National University students place a significantly higher value on self-regulated learning compared to their peers at the Egyptian universities. In summary, while self-regulated learning is generally valued by medical students across all three institutions, students at National University demonstrate a stronger preference and recognition of its importance [Table 9].

This suggests potential variations in educational culture, curriculum emphasis, or student support systems that may influence the development and encouragement of SRL skills in different university settings.

Medical students’ perceptions of the role of small group learning across three universities presented in Table 10. Students rated their agreement with statements about the importance of small group learning on a four-point scale ranging from “Not at all true of me” to “Very true of me.” Overall, most students across all universities recognized the value of small group learning, with 51.6% of Alexandria students, 57.4% of Mansoura students, and 59.7% of National University students responding, “True of me.” Additionally, National University had the highest proportion of students who rated small group learning as “Very true of me” (13.0%), compared to Mansoura (8.9%) and Alexandria (7.2%). Conversely, the combined percentage of students who disagreed with the importance of small group learning (“Not at all true” and “Untrue of me”) was highest at Alexandria (41.2%), followed by Mansoura (33.7%) and National University (27.2%) [Table 10].

**Table 10.** Distribution and comparative analysis of medical students’ perceptions on the role of small group learning at the three Universities

Role of small group learning	Alexandria University “*n* = 279”		Mansoura University “n101”		National University “235”		Total
	No	%	No	%	No	%	
Not at all true of me	23	8.2	3	3.0	7	3.0	33
Untrue of me	92	33.0	31	30.7	56	24.2	179
True of me	144	51.6	58	57.4	138	59.7	340
Very true of me	20	7.2	9	8.9	34	13.0	59
Range	1–4		1.00–4.00		1–4		
Mean	2.5		2.70		2.8		
SD	0.70		0.61		0.6		
ANOVA test	10.534						
P value	0.001*						
P1	0.05*						
P2	0.001*						
P3	0.124						

P1 comparison between Alexandria and Mansoura

P2 comparison between Alexandria and National

P3 comparison between Mansoura and National

Mean scores reflect these trends, with National University students reporting the highest mean perception of small group learning’s role (M = 2.8, SD = 0.6), followed by Mansoura (M = 2.7, SD = 0.61) and Alexandria (M = 2.5, SD = 0.70). The ANOVA test showed a statistically significant difference in perceptions among the three universities (F = 10.534, *p* = 0.001) [Table 10].

Pairwise comparisons revealed a significant difference between Alexandria and National University (*p* = 0.001) and a borderline significant difference between Alexandria and Mansoura (*p* = 0.05), while no significant difference was found between Mansoura and National University (*p* = 0.124). These results suggest that students at National University place greater importance on small group learning compared to their counterparts at Alexandria, with Mansoura students occupying an intermediate position [Table 10].

In conclusion, while small group learning is generally valued among medical students across all three institutions, the degree of appreciation varies significantly, with National University students demonstrating the strongest endorsement of its role. These differences may be influenced by variations in teaching methodologies, cultural attitudes towards collaborative learning, or institutional emphasis on small group activities.

The distribution of medical students’ perceptions regarding the impact of motivation and goal setting on their learning across three universities presented in Table 11. Students rated their agreement on a four-point scale from “Not at all true of me” to “Very true of me “. Most students across all three universities acknowledged the positive impact of motivation and goal setting on their learning. Specifically, 61.3% of Alexandria students, 74.3% of Mansoura students, and 70.1% of National University students responded, “True of me.” Additionally, the proportion of students who rated this impact as “Very true of me” was relatively consistent, ranging from 7.9% at Mansoura to 11.3% at National University. Conversely, the percentage of students who disagreed with the statement (“Not at all true” and “Untrue of me”) was highest at Alexandria (28.3%), compared to Mansoura (17.8%) and National University (18.6%) [Table 11].

**Table 11.** Distribution and comparative analysis of the impact of motivation and goal setting on learning among medical students at the three universities

Impact of Motivation and Goal Setting on Learning	Alexandria University “*n* = 279”		Mansoura University “n101”		National University “235”		Total
	No	%	No	%	No	%	
Not at all true of me	10	3.6	2	2.0	3	1.3	15
Untrue of me	69	24.7	16	15.8	40	17.3	125
True of me	171	61.3	75	74.3	162	70.1	408
Very true of me	29	10.4	8	7.9	30	11.3	63
Range	1–4		1.33 −4.00		1–4		
Mean	2.8		2.92		2.9		
SD	0.64		0.52		0.5		
ANOVA test	3.002						
*P* value	0.05*						
P1	0.021*						
P2	0.027*						
P3	0.962						

P1 comparison between Alexandria and Mansoura

P2 comparison between Alexandria and National

P3 comparison between Mansoura and National

Mean scores further illustrate these differences, with Mansoura students reporting the highest mean impact of motivation and goal setting on learning (M = 2.92, SD = 0.52), followed closely by National University (M = 2.9, SD = 0.5) and Alexandria (M = 2.8, SD = 0.64). The ANOVA test indicated a statistically significant difference among the three universities (F = 3.002, *p* = 0.05). Pairwise comparisons revealed significant differences between Alexandria and Mansoura (*p* = 0.021) and between Alexandria and National University (*p* = 0.027), while no significant difference was found between Mansoura and Sohar (*p* = 0.962) [Table 11].

This suggests that students at Mansoura and National universities perceive motivation and goal setting as having a stronger influence on their learning compared to students at Alexandria University. In summary, motivation and goal setting are generally recognized as important factors influencing learning among medical students, with Mansoura and National University students demonstrating a higher appreciation of their impact. These findings may reflect differences in academic culture, support systems, or teaching strategies that emphasize goal-oriented learning in these institutions.

The distribution of medical students’ perceptions regarding the influence of external resources on their learning across the three universities presented in Table 12. Students rated the extent to which external resources impact their learning on a four-point scale ranging from “Not at all true of me” to “Very true of me.” Most students from all three universities acknowledged the significant influence of external resources on their learning. Specifically, 47.0% of Alexandria students, 58.4% of Mansoura students, and 69.3% of National University students responded, “True of me,” while a substantial proportion also selected “Very true of me,” with Alexandria reporting the highest percentage at 34.8%, followed by Mansoura at 29.7%, and National University at 22.1%. Conversely, relatively few students disagreed with the influence of external resources, with “Not at all true of me” and “Untrue of me” responses collectively ranging from 15.3% at Alexandria to 9.1% at National University [Table 12].

**Table 12.** Distribution and analysis of the influence of external resources on learning among medical students at the three Universities

Influence of External Resources on Learning	Alexandria University “*n* = 279”		Mansoura University “*n* = 101”		National University “*n* = 235”		Total
	No	%	No	%	No	%	
Not at all true of me	13	4.7	1	1.0	1	0.4	15
Untrue of me	38	13.6	11	10.9	19	8.2	68
True of me	131	47.0	59	58.4	160	69.3	350
Very true of me	97	34.8	30	29.7	55	22.1	178
Range	1–4		1.00–4.00		1–4		
Mean	3.1		3.13		3.1		
SD	0.73		0.61		0.5		
ANOVA test	0.109						
*P* value	0.896						
P1	0.725						
P2	0.681						
P3	0.971						

P1 comparison between Alexandria and Mansoura

P2 comparison between Alexandria and National

P3 comparison between Mansoura and National

The mean scores for the influence of external resources were very similar across the three universities: Alexandria (M = 3.1, SD = 0.73), Mansoura (M = 3.13, SD = 0.61), and National University (M = 3.1, SD = 0.5). The ANOVA test indicated no statistically significant difference among the universities regarding this perception (F = 0.109, *p* = 0.896). Pairwise comparisons further confirmed the lack of significant differences between Alexandria and Mansoura (*p* = 0.725), Alexandria and National University (*p* = 0.681), and Mansoura and National University (*p* = 0.971) [Table 12].

This suggests a consistent recognition across all institutions of the important role that external resources play in supporting medical students’ learning. In conclusion, external resources are widely regarded as vital contributors to medical education by students at Alexandria, Mansoura, and National universities. The uniformity in responses highlights a shared understanding of the value of supplementary materials such as online databases, videos, textbooks, and medical websites across diverse educational contexts.

## Discussion

This multi-institutional cross-sectional study provides valuable insights into the learning preferences and behaviors of medical students across three universities in Egypt and Oman, highlighting important variations and commonalities that have significant implications for medical education. Our findings demonstrate that while students universally recognize the importance of blended learning modalities, self-regulated learning, and external resources, distinct differences exist in the degree of engagement and preference for specific learning strategies and tools.

Notably, students at National University consistently exhibited stronger preferences for online resources, small group learning, and self-regulated learning compared to their counterparts at Alexandria and Mansoura Universities. These differences likely reflect variations in institutional culture, resource availability, and pedagogical approaches, underscoring the need for tailored educational interventions that address the unique contexts of each institution. Understanding these nuanced preferences is crucial for optimizing curriculum design, enhancing student engagement, and ultimately improving learning outcomes in medical education. This discussion will explore these findings in depth, situating them within the broader literature and offering evidence-based recommendations for educators and policymakers.

The study involved medical students in different age groups, about 52% aged 17–20 and about 44% aged 20–25 years and only 4% aged 25–27 which is typical for undergraduate medical students [16]. The students from Alexandria University were younger (mean age 19.5 years) because students in Egypt typically begin medical school at an earlier age just after graduation from high school [17], whereas students at National University were older (mean age 20.8 years) due to 1 year foundation between high school and medical school and anatomy is taught over one year only. While in Mansoura university, the mean age was 23.6 years because anatomy is taught through the module system till the third year.

There were more females than males included in this study (61%). There were no gender differences in learning preferences regarding online learning, face to face lectures or BL indicating that all modalities are equally valued by both male and female students. Nevertheless, female students showed a significant preference for small group education (*p* = 0.003), which is in line with previous reports that female students are more likely to participate in interactive learning activities [18].

This study reflects the differences in the preferred online tool for anatomy learning used by the students from the three universities. Alexandria and Mansoura students prefer Telegram. Telegram is a widely used platform in the medical schools in Alexandria and Mansoura Universities, where professors, lecturers and senior students share their educational resources, which are easily communicated with the students. National University students prefer You tube, Google, online books, Instagram and medical websites. LMS Moodle was valued highest by National University students as the lectures are uploaded on a specific Moodle (Black Board). YouTube is a multimedia platform famous for its rich content, favored by students due to its accessibility and ease of use [19].

These variations in preferred online tools suggest variable institutional resources, environmental and cultural factors that may significantly influence students’ choices regarding online learning platforms. This reflects the curricular approaches and the strong institutional support for such platforms, as well as the familiarity of the students with these resources in their learning environment. These differences emphasize the importance of understanding local cultural and environmental contexts when designing and implementing learning strategies in diverse educational settings.

The results of the study highlight significant differences in student preferences for learning modalities across the three universities. Students at the 3 Universities prefer BL and online learning over face-to-face lectures. This preference may be due to the availability and familiarity of online resources in their learning environment, as well as the increasing trend toward digital education in these Universities. Recent evidence indicates that students surrounded by an expanding online learning environment tend to favor online learning due to its more resilience and conveniency [20]. The preference for online learning can also be explained by the impact of the COVID-19 pandemic. Introduction of online modalities in these universities resulted in increased student familiarity and comfort with online learning as was the case in many other universities around the globe during the pandemic [21].

Students at National University Showed the highest preference for face-to-face lectures suggesting that while they appreciate the resilience of online learning, they still value the interactivity of traditional lectures. This blended preference is consistent with the notion that BL offers the best of both modalities, providing students with both flexibility of online learning and the opportunity for direct interaction with educators [22].

Students’ preference for face-to-face lectures at National University can be explained by the fact that the number of students in any one patch is less, and the lecturers are the main source for scientific material in the form of power point presentations and lab demonstrations. This is consistent with previous reports from studies in the Gulf countries, where students may place higher value for direct interaction with instructors, particularly in medical education, where mutual discussions and practical training can take place [23]. The overwhelming preference for face-to-face lectures in National University could also be attributed to the assessment methods, where in-person learning and direct engagement with teachers is essential particularly for complex subjects such as anatomy [24].

One of the major advantages of BL for medical students is the flexibility and convenience of online learning and it was valued highly by students from the 3 universities (mean = 3.26 for National, 3.1 for Alexandria and 2.97 for Mansura). Online learning improves the time management skills of the students, advances them into SRL and decreases the workload on the faculty [25].

The results indicated that students across the 3 universities prefer external audio-visual resources (mean = 3.25 for Mansoura, 3.23 for National University and 3.2 for Alexandria). This proves that all students in different learning environments benefit more from online resources, especially the audiovisual tools which reinforce learning of anatomy and are crucial for understanding complex anatomical relations through audiovisual presentations [26, 27]. Students at National University value the audiovisual resources provided by their college very highly (mean = 3.06). This has been a point of weakness in the past where audiovisual resources provided by the college were scarce. Recently this has been adjusted by the provision of many online programs like 3D anatomy and complete Anatomy.

Small group learning such as case-based learning (CBL), problem-based learning (PBL) and team-based learning (TBL) enhance active learning and encourage peer collaboration [28]. This learning modality was more favored by National University students (mean = 2.8) and Mansoura students (mean = 2.7) and to a less extent by Alexandria students (mean = 2.5). The increased rating for small group learning in National and Mansura Universities reflects the collaborative culture in these universities, where peer learning and interactive sessions are highly valued. This can also be explained by the small number of students in one patch and the majority of female students at National University.

Students at the 3 universities showed high tendency and preference for SRL especially in National University (mean = 3.1) followed closely by Alexandria (mean = 2.9) and Mansoura (mean = 2.82) universities. This is due to the availability of the learning material, easy access to online resources, convenience and resilience of teaching modalities in the 3 universities. One of the main targets of medical education in any medical school is to help students to become self-regulated lifelong learners [29].

The great engagement with BL modalities in the three universities suggests that institutional pedagogy plays a significant role in shaping students’ learning behaviors and attitudes [30]. At Alexandria University, where a more balanced approach between online and face-to-face lectures is evident, there is greater institutional support for blending different teaching methods. In contrast, the higher preference for online learning at Mansoura University reflects the strengths of the educational infrastructures and the mature independence of the students. The preference of face-to-face learning at National University reflects the unique cultural and environmental factors in Oman, where assessments methods depend largely on the material provided in the lectures. These variations should be taken into consideration during curriculum development and implemented strategies should be tailored to meet the specific needs of students at each university.

The influences of group discussions and peers on study habits were rated lowest by students in the 3 universities. This suggests that students in these institutions may prefer independent or individualized study methods. Therefore, while small group work can be valuable for reinforcing learning, its impact might be less pronounced in environments where traditional study habits dominate [31]. 

Our study revealed that the National university demonstrated a stronger preference for various BL modalities compared to Alexandria and Mansoura universities. Several institutional factors may explain these differences, offering insights into best practices for anatomy education with BL.

National university is the only private medical school among the three, operating with an outcome-based 7-year Doctor of Medicine program accredited by TEPDAD (Association of Medical Education Programs Evaluation and Accreditation). The program integrates premedical, preclinical, and clinical years within a learner-centered environment emphasizing experiential learning, critical thinking, and lifelong learning. National university’s adoption of Blackboard as its sophisticated Learning Management System (LMS), coupled with extensive IT infrastructure—such as high-specification computer labs and high-speed Wi-Fi access across campus—provides students with seamless access to online resources [32]. 

The curriculum in National university is outcome-based where anatomy is taught separately while in Alexandria and Mansoura universities, it is system-based where anatomy becomes a part of the module. The structure of the anatomy courses in National university is more convenient for the students, where anatomy is divided into 4 courses, anatomy I for general anatomy, embryology and histology, anatomy II for upper and lower limbs, thorax, abdomen and pelvis, anatomy III for head and neck and anatomy IV for Neuroanatomy. This strong technological and pedagogical support likely contributes to students’ higher valuation of SRL. National university’s focus on bridging preclinical and clinical learning (vertical integration) may also enhance student engagement with blended modalities [32]. 

Alexandria Faculty of Medicine (AFM) offers a competency-based 5-year MBBCH program aligned with the Egyptian National Academic Reference Standards (NARS). The curriculum is structured into two phases—pre-clerkship and clerkship—with anatomy education concentrated in the first four semesters using integrated systems-based blocks. Alexandria uses Moodle as its LMS, providing a platform for online content delivery but with less extensive technological infrastructure compared to National university. The integrated curriculum design and concentration of anatomy teaching in early semesters may influence student preferences for specific modalities, such as face-to-face or small group sessions, during foundational years [33]. 

Mansoura Faculty of Medicine transitioned from a traditional 6-year discipline-based curriculum to a fully integrated modular 5-year program. This approach merges basic and clinical sciences into modules that promote active student participation through diverse teaching methods. Like Alexandria, Mansoura uses Moodle as its LMS. The modular, integrated structure aims to foster deeper understanding through various interactive strategies, which may explain nuanced preferences in BL modalities among its students [34]. 

Regarding specific modality use, all three institutions employ face-to-face lectures primarily during core topics but vary in their extent. Alexandria emphasizes face-to-face and small group anatomy teaching during the pre-clerkship phase. Mansoura adopts modular-based sessions blending different teaching techniques, while National university combines traditional lectures with extensive online and experiential learning throughout all years. These curricular distinctions shape students’ preferences for face-to-face, online, and BL, underscoring the importance of contextualizing BL strategies within institutional frameworks.

In sum, accounting for differences in curriculum structure, LMS platforms, technological resources, and pedagogical approaches helps explain the variability in BL preferences observed. Best practices may include enhancing technological infrastructure, fostering SRL, and integrating flexible, student-centered blended modalities tailored to each institution’s curricular context.

These findings highlight the varying educational preferences among students in the 3 universities, influenced by institutional contexts, variable educational infrastructures and suggest that tailored approaches for future curriculum development strategies may enforce the exceptional strengths of each institution and enhance learning experiences across different universities.

Garrison & Vaughan, 2008 emphasize that educational strategies must be aligned with student preferences to maximize learning outcomes. Medical schools should focus on utilizing BL approaches that are applicable, adoptable, and responsive to student needs, ensuring that students grasp the necessary skills to become lifelong learners and adapt to developing medical knowledge and professional environment [35]. 

### Limitations of the study

While this study offers valuable multi-institutional and cross-cultural insights into medical students’ learning preferences and behaviors within blended learning environments, several limitations should be considered. First, the cross-sectional design provides a comprehensive snapshot of student perspectives at a specific point in time, allowing for meaningful comparisons across institutions. However, it inherently limits the ability to establish causal relationships between learning preferences and academic outcomes. Future longitudinal studies could build on these findings to explore how preferences evolve and impact performance over time.

Second, the study relies on self-reported data, which may be subject to response biases such as social desirability or recall inaccuracies.

Third, although the sample includes a substantial number of students from three universities in two different countries, it may not fully represent the broader population of medical students in the region. Institutional and cultural factors unique to these universities could influence the generalizability of the results.

Finally, the quantitative survey methodology enabled efficient data collection and statistical analysis across a large sample but may have limited the depth of understanding regarding the nuanced motivations and contextual factors influencing learning preferences. Incorporating qualitative approaches in future research could enrich interpretation and provide more detailed insights.

In summary, while these limitations are acknowledged, the study’s strengths include its multi-institutional design, robust sample size, and systematic comparison of key learning constructs provide a solid foundation for advancing understanding in medical education. The findings offer actionable insights for educators and policymakers aiming to tailor BL approaches to diverse student populations, and they pave the way for future research to build upon and deepen this knowledge.

## Conclusions

This study provides a comprehensive multi-institutional perspective on medical students’ learning preferences and behaviors within blended learning environments across diverse cultural and educational contexts. The findings highlight that while students universally value blended learning modalities, significant variations exist in their engagement with online resources, small group learning, self-regulation, and motivational strategies.

Notably, students at National University demonstrated stronger preferences for self-regulated learning and the use of diverse digital tools, underscoring the influence of institutional culture and resource availability on learning approaches. Gender differences were minimal overall, except for a pronounced female preference for small group education, suggesting the need to consider gender-responsive pedagogical strategies.

The consistent recognition of external resources’ importance across all universities emphasizes the critical role of supplementary materials in modern medical education. These insights highlight the necessity for medical educators and curriculum designers to adopt flexible, learner-cantered approaches that accommodate varied preferences and foster effective self-regulated learning. Tailoring educational strategies to the unique needs and contexts of each institution can enhance student motivation, engagement, and ultimately, academic success. Future research should build on these findings by exploring longitudinal outcomes and integrating qualitative perspectives to deepen understanding of the complex dynamics shaping medical students’ learning experiences.

## Data Availability

The data sets collected and analyzed during this study are available from the corresponding author on appropriate request.

## Abbreviations

BLQ: Blended Learning Questionnaire
BL: Blended Learning
SRL: Self-regulated learning
